# Leadership matters: a systems approach to strengthening the quality of child and youth mental health care

**DOI:** 10.1093/eurpub/ckaf242

**Published:** 2026-01-23

**Authors:** Jennifer Hall, Hannah Brunskill, Válter R Fonseca, Anastasia Gatopoulou, Anastasia Giannaki, Penny Kalpaxi, Elena Maousidi- Zirganou, Raffaella Sibilio, Shobhan Thakore, Ledia Lazëri, João Breda

**Affiliations:** WHO Office on Quality of Care and Patient Safety in Athens, WHO Regional Office for Europe, Athens, Greece; WHO Office on Quality of Care and Patient Safety in Athens, WHO Regional Office for Europe, Athens, Greece; WHO Office on Quality of Care and Patient Safety in Athens, WHO Regional Office for Europe, Athens, Greece; WHO Office on Quality of Care and Patient Safety in Athens, WHO Regional Office for Europe, Athens, Greece; WHO Office on Quality of Care and Patient Safety in Athens, WHO Regional Office for Europe, Athens, Greece; WHO Office on Quality of Care and Patient Safety in Athens, WHO Regional Office for Europe, Athens, Greece; WHO Office on Quality of Care and Patient Safety in Athens, WHO Regional Office for Europe, Athens, Greece; WHO Office on Quality of Care and Patient Safety in Athens, WHO Regional Office for Europe, Athens, Greece; Scottish Quality and Safety Fellowship Programme, NHS Education for Scotland, Edinburgh, Scotland; Mental Health and Well-being, WHO Regional Office for Europe, Copenhagen, Denmark; WHO Office on Quality of Care and Patient Safety in Athens, WHO Regional Office for Europe, Athens, Greece

Strengthening the quality of child and youth mental health (CAYMH) care is a priority across the WHO European Region [1]. In response, much activity has been underway in the WHO Regional Office for Europe, including the setup of a dedicated workstream and the development of the WHO Quality Standards for CAYMH Services [2].

Despite these efforts, many challenges to providing high-quality care for CAYMH exist across the Region. In this paper, we outline some of these challenges, situate them within the wider framework of complex and adaptive systems, and consider how engaging with leaders can help to overcome some of these challenges.

## Systemic challenges to providing high-quality CAYMH care

Countries across the Region are facing similar challenges to providing high quality CAYMH care, some of which are outlined below:

1. Increase in population need for CAYMH services: The number of children and adolescents aged 0–19 years old living with mental health conditions has increased by approximately one third over the past 15 years, and over one in five adolescents aged 15–19 years old lives with a mental health condition [3]. CAYMH services are receiving more referrals, leading to longer waiting lists and the need to prioritize which children and young people receive care.
2. Changing environments: Children and young people are exposed to new stressors, such as climate change, escalating conflict and navigating unregulated digital environments [1]. This leads to the need to manage these emerging issues before adequate evidence has been generated.
3. Understaffing: Staff shortages are seen in most CAYMH services across the Region [3]. When services are not adequately staffed, there is not only a lack of ability to implement high-quality care (e.g. due to lack of specialists), but also an increased risk of burnout, which leads to lower quality of care.
4. Under funding: CAYMH services often do not receive adequate financial support [4]. Resources are needed to create high-quality services [2], for example to set up services in community settings, work across multiple sectors, provide training to staff and set up structures for meaningful participation of users.
5. Lack of evidence on how to improve quality of CAYMH care: Initiatives to improve quality of care and patient safety within CAYMH care are centered only in a few countries, and research into their effectiveness is limited [5]. The application of findings from other health areas to CAYMH may be limited.

## Complex systems thinking as a possible solution

These challenges are all unified by being complex in nature. As such, these challenges should not be considered as isolated “cogs” in a machine, but instead as part of a wider complex public health system of “mechanisms, dynamics and patterns” [6] which impact on CAYMH outcomes, and within which, interventions can have unforeseen consequences.

To meet the holistic needs of children and young people with mental health conditions, it is required to work within health systems and across a wide range of different sectors and systems (e.g. families, education, child protection). Hence, overcoming these challenges not only needs consideration of health systems, but also consideration of broader societal and political systems across multiple sectors, across a complex eco-system of care.

## Leaders are pivotal to strengthen the quality of care

The onus to solve complex challenges and strengthen the quality of care often falls on health managers and clinicians [7]. This is also true for CAYMH, hence it is essential to engage with health managers and clinicians to improve quality across the system.

For a whole-of-systems approach to improving quality of care, advocacy and influence from communities and users of services is also needed [8]. To allow for this, we push for leaders in CAYMH services to meaningfully engage stakeholders across the ecosystem of care in all decisions that affect them. This includes—but is not limited to—teachers, health workers, policymakers, children, adolescents, young people and families.

## How to best support leaders to strengthen quality of CAYMH care

To improve health systems performance, skills in human factors, service design, systems thinking, quality improvement and leadership are recommended [9]. Leadership competencies for improving quality of care include expertise (quality improvement, health area), ability to motivate others, set a vision, communicate, and be supportive and trustworthy [7].

However, those working in leadership positions in health systems are often trained only in approaches to diagnose and manage health problems. This is especially true for those working in CAYMH, where no training curriculum to improve the quality of CAYMH services was found to exist [10], and evidence on the impact of capacity building and leadership on the quality of CAYMH services is lacking [5].

## A course to support health leaders to improve the quality of CAYMH care across the WHO European region

Without a pre-existing training curriculum for improving the quality of CAYMH services, inspiration can be taken from other capacity-building efforts and tailored for common challenges faced by those working in CAYMH services.

The proposed curriculum echoes previous trainings [9] and includes quality improvement, human factors, service design, systems thinking and leadership. More specifically and tailored to the needs of those working in CAYMH services, the curriculum includes a focus on innovative and creative solutions to overcome common challenges (e.g. design thinking, sharing of ideas), how to reduce the risk of burnout in understaffed teams, how to apply learnings to the ecosystem of care for CAYMH, and how to meaningfully engage with communities and young people with lived experience.

In addition to these technical skills, the curriculum also includes a focus on joint problem solving, peer learning and building a strong community of practice. To support this, methods to deliver training facilitates psychological safety, collaboration and peer learning.

## Discussion

Leaders working in CAYMH services are tasked with solving complex problems, often without adequate training or support to do so. By engaging with these leaders, it is possible to enable them to understand the nature and dynamics of complex and adaptive systems and change management, to work for better quality CAYMH systems. One way to achieve this is through targeted capacity-building initiatives in the aforementioned curriculum.

A training course alone is not sufficient. Leaders’ ability to implement the learnings may depend on the setting in which they work, highlighting the need for wider institutional support, ongoing supervision and peer learning. A balanced approach needs to be taken to ensure that the capacity strengthening does not inadvertently add undue pressure to already high workloads, increasing the risk of burnout and compromising quality of care.

Engaging with leaders should be part of a whole-of-system approach to improve quality of CAYMH care, including supportive policies and legislation, treatment guidelines, and increased evidence.

We call for efforts to build leadership capacity and create enabling environments that empower leaders to overcome complex systemic challenges to delivering high-quality CAYMH care. However, this is just one piece of the puzzle. More coordinated action is needed across all levels of the eco-system of care to improve outcomes for CAYMH, to build stronger and more resilient communities, for now and in the future.

Conflict of interest: There are no conflicts of interest to declare.

## Data Availability

Data sharing is not applicable to this article as no datasets were generated or analyzed.
